# Comparative Transcriptomics Between Zebrafish and Mammals: A Roadmap for Discovery of Conserved and Unique Signaling Pathways in Physiology and Disease

**DOI:** 10.3389/fcell.2019.00005

**Published:** 2019-02-01

**Authors:** Huma Shehwana, Ozlen Konu

**Affiliations:** ^1^Department of Molecular Biology and Genetics, Bilkent University, Ankara, Turkey; ^2^Department of Multidisciplinary Studies, National University of Medical Sciences, Rawalpindi, Pakistan

**Keywords:** zebrafish, transcriptomics, mammals, cancer, toxicology, development, physiology, comparative

Zebrafish is a small fresh-water species widely used as a vertebrate model in human physiology and pathologies. In addition, the zebrafish genome has recently been sequenced providing a plethora of opportunities for comparison with the genomes of other animals, such as rodents and humans (Howe et al., 2013). However, there is an emerging need for a concise synthesis of the literature findings and approaches used in comparing transcriptomes of mammals with that of zebrafish. In this opinion article, we first focus on features which make zebrafish genome and transcriptome unique and invaluable for comparative studies. Exploring research findings under multiple topics such as comparative physiology, toxicology, and cancer reveal important aspects of functional conservation among vertebrates and effective research pipelines for comparative transcriptomics studies.

## Features of Zebrafish Genome/transcriptome

The zebrafish genome is similar to the human genome with respect to the number of chromosomes (25 vs. 23 pairs, respectively) (Postlethwait et al., 2000) and of protein coding genes (Howe et al., 2013). On the other hand, a distinguishing aspect of the zebrafish genome, as that of most fish genomes, is the extra whole-genome duplication (WGD) event that has been estimated to occur at least 320 million years ago (Hoegg et al., 2007). As a result, the zebrafish genome contains paralogous pairs of genes, exhibiting divergent or complementary functions, for a set of mammalian orthologous genes (Laprairie et al., 2016). The divergence of zebrafish paralogous genes with respect to their methylation pattern in correspondence to expression levels in gametes and early embryos has been studied (Zhong et al., 2016). Two thousand four hundred and forty paralogous pairs of genes have been identified and more than 75% of these genes are found to be under purifying selection. In addition, around 600 duplicate pairs in zebrafish exhibit divergent promoter methylation as well as a negative correlation between levels of promoter DNA methylation and mRNA expression (Zhong et al., 2016). Future studies are still needed to better understand the expression patterns of zebrafish paralogs in comparison to human orthologous genes. In this context, we are developing a web-based tool called *CompariZome* for comparative statistical examination of public human and zebrafish expression datasets extracted from GEO repository (Edgar et al., 2002; Lopes et al., 2016).

## Identification of Conserved Functional Processes by Comparative Transcriptomics

**Figure 1:**
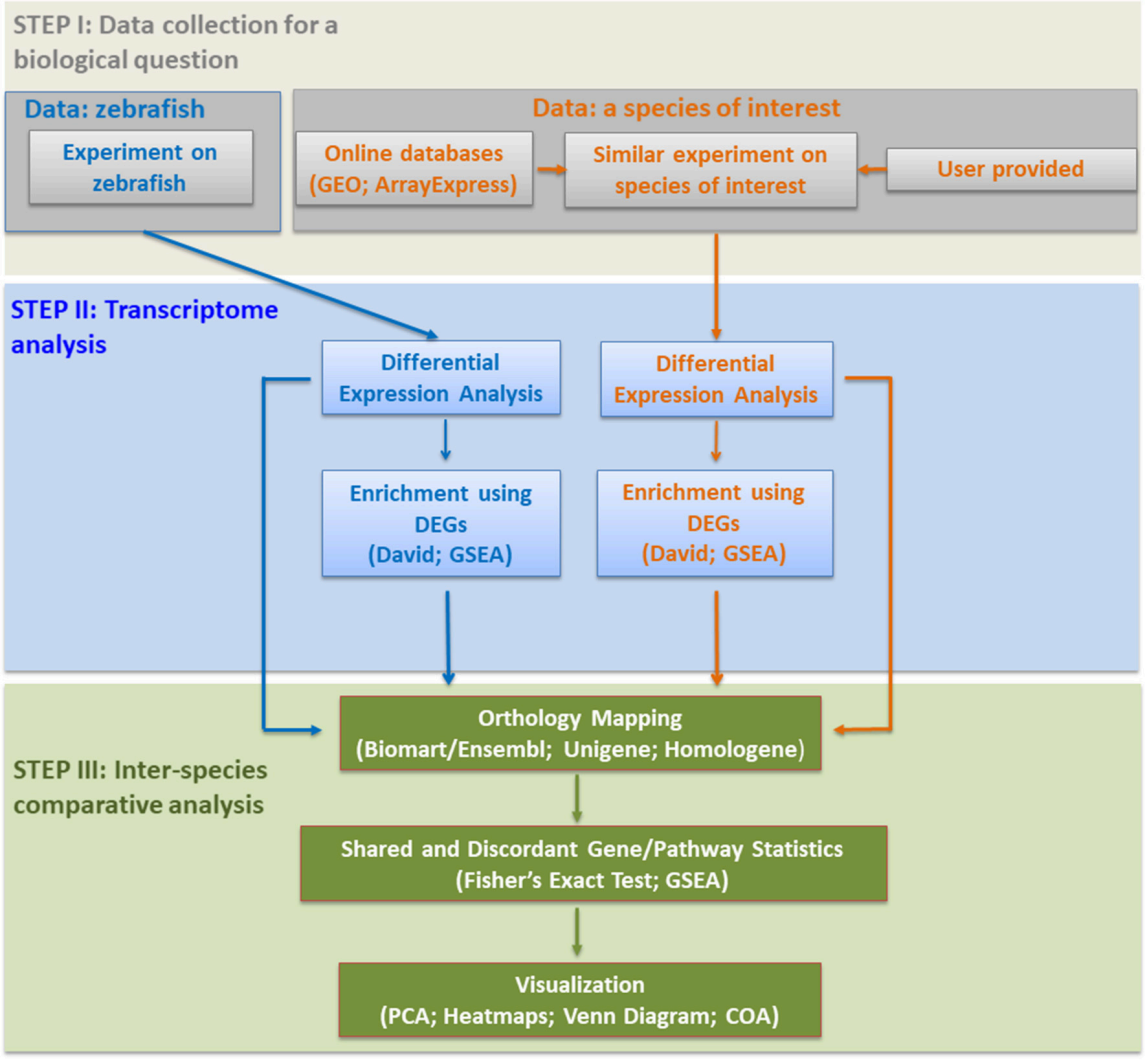
A research pipeline for comparative transcriptomics between zebrafish and mammals.

Based on previous studies, two effective and complementary approaches exist for comparative transcriptomics between different species: comparison of significantly modulated (a) genes; and/or (b) pathways (Figure 1). We briefly explain them below with selected examples from the literature.

For any comparative study dealing with zebrafish and mammalian expression, multiple data sources (Edgar et al., 2002; Kolesnikov et al., 2015) need to be searched for a specified biological question (Figure 1). Upon obtaining differentially expressed genes (DEGs), the next crucial step is to identify the pathways enriched in the gene lists of each species, separately. A commonly used web-based software is DAVID (Huang da et al., 2009a,b; Schartl et al., 2012; Sucularli et al., 2016), used to identify the enriched functional terms, e.g., KEGG (Kyoto Encyclopedia of Genes and Genomes) pathways or gene ontology (GO) (Figure 1). Gene Set Enrichment Analysis (GSEA), which was developed to rank significantly enriched biological/cellular processes based on differentially expressed genes (Subramanian et al., 2005), is also widely used (Zheng et al., 2011; Yildiz et al., 2013). One of the most important steps in comparative transcriptomics is orthology mapping (Figure 1). This can be performed using different resources that include Ensembl Biomart (Saraiva et al., 2015; Shih et al., 2015); Unigene clusters (Lam et al., 2006; Zheng et al., 2011, 2014); and Homologene (Driessen et al., 2015). Statistics based on Fisher's Exact test (Sucularli et al., 2016) or GSEA (Zheng et al., 2011) are essential elements to reveal significant associations between different species in terms of the selected genes, pathways or enrichment terms (Figure 1). Moreover, the visualization of such associations between different species requires the use of multivariate exploratory techniques among which Principal Component Analysis (PCA) and heatmaps (Zheng et al., 2014; Saraiva et al., 2015; Tarifeño-Saldivia et al., 2017), and/or coinertia (COA) plots (Kaya et al., 2011) are commonly preferred (Figure 1). Representation by Venn diagrams is another way to show the intersection of gene lists/pathways between species (Lam et al., 2006; Tarifeño-Saldivia et al., 2017). The abundance of one-to-many orthology relationships between zebrafish and mammals, however, complicates orthology mapping and hence may restrict downstream analyses to the use of only one-to-one orthologous pairs that exhibit moderate-high sequence similarity (Saraiva et al., 2015; Davison et al., 2017; Tarifeño-Saldivia et al., 2017). Finally, the development of online tools and use of meta-analysis methodologies can lead to easier access to data/analyses and more robust conclusions on the degree of functional conservation between zebrafish and mammals (Kaya et al., 2011; Sucularli et al., 2016; Davison et al., 2017).

To demonstrate different aspects of the pipeline described in Figure 1, we provide the below examples of comparative transcriptomics studies focusing on physiology, toxicology, and cancer.

## Physiological Processes and Disease Relevance

Inter-species transcriptome analysis is highly useful to discover common, as well as unique, signatures in a diverse set of physiological and relevant pathological conditions. For example, RNA-sequencing of zebrafish heart has led to the identification of 96 chamber-specific genes, 68 of which possess orthologs in humans. An OMIM database search reveals the 25 of these 68 genes are disease-associated, and five of them are specifically involved in cardiac diseases (Singh et al., 2016). Similarly, 49 orthologs of 51 known human dilated cardiomyopathy-associated genes are found to be conserved in zebrafish, further promoting the use of the zebrafish model for human cardiac disease research (Shih et al., 2015). However, 19 of these genes have two or more paralogs in zebrafish, pointing to one of the complexities that needs to be resolved for studying disease association between zebrafish and humans. Currently, the selection of a gene that is enriched within the specified tissue emerges as a potential strategy to prioritize one or more of the paralogous genes for future studies (Shih et al., 2015).

Maternal to zygotic transition (MZT), during which maternally provided RNA and proteins are replaced by those produced by the zygote alone, is a shared phenomenon among animals (Bensaude et al., 1983; Harvey et al., 2013). The comparison of expression changes in MZT of *Lymnaea stagnalis* with the published data from several arthropod, nematode, urochordate, and chordate species including zebrafish, has indeed led to the extraction of a shared expression signature. An analysis of orthologs common to all species (identified using tBLASTx) reveals that maternal transcripts are often enriched with housekeeping functions and are involved in nucleotide binding, cell cycle progression, and protein degradation (Liu et al., 2014). This extensive comparative transcriptomics study of embryogenesis shows the power of such an approach and contributes to our understanding of the patterns of embryonic conservation among vertebrates and invertebrates. Future comparative transcriptomics studies between zebrafish and mammals can help reveal the functional divergence of paralogous genes in zebrafish MZT.

Other areas receiving attention in comparative transcriptomics include regulation of the circadian clock between zebrafish and mouse (Boyle et al., 2017) and the degree of conservation of ovulation in humans, mice, and zebrafish (Liu et al., 2017). Zebrafish has also become a useful model for revealing the resemblance between zebrafish swim bladder and mammalian lung (Zheng et al., 2011), renal distal convoluted tubules of zebrafish and mouse (Sugano et al., 2017), pancreatic cell populations of zebrafish, mouse, and human (Tarifeño-Saldivia et al., 2017), and olfactory systems of mouse and zebrafish (Saraiva et al., 2015). In addition, the conservation of immune response between zebrafish and mammals has been shown using meta-genomics of gut microbiota (Davison et al., 2017) and with respect to IFN gamma signaling (Filiano et al., 2016).

## Cancer

Cancer, one of the complex human diseases threatening large numbers of people across different age groups involves the deregulation of expression of genes relevant to cell proliferation, apoptosis, and senescence (Jones and Baylin, 2007). Hence, understanding how similarly genes and signaling pathways are modulated between zebrafish and humans can help identify novel therapeutic gene/protein targets and signaling pathways in cancer.

For example, comparisons between medaka, zebrafish, and human melanoma transcriptomes using RNA sequencing have identified more downregulated than upregulated genes that are commonly modulated between fish and human samples (Schartl et al., 2012). Another study focusing on the comparison of genetic alterations in zebrafish and human melanoma (exhibiting congruent BRAF mutation and P53 deletions) points to lower rates of UV-induced mutations in zebrafish while providing novel candidate genes to pursue in drug-resistant human melanomas (Kansler et al., 2017). Similarly, a study performed with an inducible zebrafish embryonal rhabdomyosarcoma model has identified the shared upregulation of *MYF5* mRNA in zebrafish and human expression datasets based on GSEA comparisons (Langenau et al., 2007). Further transcriptomics and mechanistic studies in zebrafish and human rhabdomyosarcomas have demonstrated the potent role of the transcription factors MYF5 and MYOD in the development and growth of muscle tumors (Tenente et al., 2017).

Using statistical approaches, such as binomial tests and GSEA has shown that zebrafish liver cancer most significantly resembles human liver cancer in terms of changes in gene expression (Lam et al., 2006). Further studies demonstrate that deregulations in several cancer-related transcription factors, MYC, E2F1, YY1, and STAT, are commonly observed in zebrafish and human liver cancers (Ung et al., 2009). It has also been shown that different human liver cancer subtypes can be paired successfully with *xmrk-, kras-*, and *myc*-induced zebrafish tumors using comparative transcriptomics at the level of gene as well as at the level of cellular pathways (Zheng et al., 2014). The authors have found that the human orthologs of the genes upregulated in zebrafish liver tumors are also upregulated in different stages of hepatocellular carcinomas (HCC); and altogether, three types of zebrafish models represent almost half of the human HCC samples (Zheng et al., 2014).

## Toxicology and Pharmacology

Toxicology and pharmacology are the two relevant fields in which the comparison of zebrafish and mammalian transcriptomes provide invaluable information for translational biology. Liver cells, mainly due to their ability for detoxification, are frequently expression-profiled in human toxicology studies where comparisons with zebrafish liver or embryos/larvae exposed to drugs are made (Driessen et al., 2015). For example, mercury can induce early and late response genes *in vivo* in zebrafish liver and *in vitro* in human liver carcinoma cells. In addition to the identification of concordant upregulated (proteasome and DNA damage response) and downregulated (mitochondrial fatty acid beta oxidation, electron transport chain, nuclear receptor signaling) pathways, this study also reveals discrepancies between transcriptomes of zebrafish and humans in response to mercury, signifying caution in such comparative studies (Ung et al., 2010). Gene-centric analyses in toxicology also exist; for example, H2O2 exposure in zebrafish larvae and in human keratinocytes shows that the expressions of 41 genes are modulated in common between the two species (Lisse et al., 2016). Scatterplots of logarithmically transformed fold expression changes (LogFC) from different expression datasets help test similarity in expression profiles between experiments and have been used in recent comparative transcriptomics literature (Yildiz et al., 2013; Lisse et al., 2016).

Zebrafish is a highly preferred model for testing drugs commonly used in treating human diseases. For example, a study performed with the immunosuppressant drug, cyclosporine, at the levels of gene, pathway and transcription factors, strengthen the confidence on the effective use of zebrafish embryos for comparative toxicogenomic studies (Driessen et al., 2013). Pathway-focused comparative transcriptomics analysis of rapamycin exposure, *in vitro*, between a zebrafish embryonic fibroblast cell line and five mouse cell lines also demonstrate the presence of a strong and positive inter-species association between expression profiles (Sucularli et al., 2016). Furthermore, the comparison of pathways/gene ontology terms via meta-analysis pipelines, as well as the stringent use of association tests, can be listed among the strategies to increase the robustness of findings in comparative transcriptomics (Sucularli et al., 2016). Using *in vivo* models in addition to *in vitro* comparisons may further lead to more comprehensive findings since a larger number of shared genes and stronger enrichment scores can be uncovered with *in vivo* studies (Driessen et al., 2015).

## Conclusion

Herein, we have provided a brief pipeline from hypothesis testing to the visualization of results for comparative transcriptomics studies between zebrafish and other animals based on the existing literature (Figure 1). We also reiterate the need to focus on the employment of effective strategies to deal with paralogous gene expression in comparative studies. Moreover, the use of meta-analysis in comparative transcriptomics is likely to increase the robustness of conclusions in regard to the functional conservation of genes and pathways between zebrafish and mammals. Similarly, the development of web-based analysis tools will enhance accessibility to comparative transcriptomics data and will allow researchers to make better decisions while selecting genes/pathways to target for further mechanistic studies or translational research.

## Author Contributions

HS and OK contributed to the ideas and the literature search of the manuscript, wrote the manuscript, and revised it.

### Conflict of Interest Statement

The authors declare that the research was conducted in the absence of any commercial or financial relationships that could be construed as a potential conflict of interest.
